# Remarks on Dr. Harvey's Practising Midwifery

**Published:** 1812-06

**Authors:** 


*To the Editors of the Medical and Physical Journal.*

GENTLEMEN,

THE authority upon which I inserted the name of *Harvey* among the early practitioners of midwifery, (see p. 440) was that of *Dr. Denman:* than whom, I know no man more conversant with the history of midwifery in England, or with the biography of its professors. In the preface to his "Introduction to the Practice of Midwifery," 4to. p. 16, he says, "By inclination, or the necessity of his affairs, *Harvey was engaged in the practice of midwifery,* by which means he got that information which enabled him to write his *Exercitatio de Partu,* and the many excellent observations upon that subject with which his works abound."

I have consulted such memoirs of the life of Harvey as I have at hand,* but have not found in them any confirmation of this opinion. I cannot doubt, however, that *Dr. Denman* had sufficient evidence of the fact when he made the assertion, and to him I must leave it to give a further answer to your correspondent, should it be thought necessary.

By an examination of *Harvey's Exercitationes de Generatione Animalium, quibus accedunt quædam de Partu, &c.* your correspondent may be satisfied, that, if not actually *engaged* in the practice of midwifery, as we understand by

---

* In the General Dictionary, folio, vol. 6, prefixed to his works published by the College of Physicians, 1766; in Aikin's Biographical Memoirs of Medicine; in Mangeti Bibliotheca Script. Medicorum; in Hutchinson's Biographia Medica, (a most unsatisfactory book of reference.)

that that expression at the present day, he was at least frequently consulted about uterine diseases, and an occasional attendant in the lying-in chamber; and that he gained information of the state of these parts, and of the progress of the labor, as our modern accoucheurs do, *Digitis explorando per vaginam.* I shall quote some passages, in support of this opinion, taken from the quarto edition of his works, published by the College, in 1766.

"Ego non arbitror fœtum—eandem semper posituram servare. Quippe in aqua natans seseque movens modo huc modo illuc extenditur, varieque inflectitur et volutatur; adeo ut proprio umbilici funiculo implexus mire interdum irretiatur."—P. 541.

"Novi juvenculam quæ ob summum pariendi laborem in animi deliquium incidit; statimque adeo consternata stupida et comatosa facta est ut nullis remediis excitari potuerit. Ad eam vocatus—pennam sternutatorio fortiori illitam in nares indidi—quoties cunque autem stimulo hoc usus sum promovebatur partus."—P. 554.

"Talem uteri constitutionem *ante partum reperi.*"—P. 565.

"Si vel sanguinis grumi, vel aliud quid præternaturale post partum in cavitate uteri remanserit; hic nec sursum retrahitur nec orificium suum claudit; sed cervix ejus mollis et aperta persentiscitur. Ut *expertus sum* in muliere——*Uteri ejus orificium laxum molle et patentissimum deprehendi,*—unde subesse aliquid in utero quod putresceret suspicatus sum; *digitisque immisis* molam—*extraxi.*"—P. 566.

"*Observavi* autem in nonnullis *orificium uteri statim à partu constrictum.*"—P. 567.

"—— Matricis ejus orificium durum et clausum, instrumento ferreo immisso, per vim aliquantulum aperui."—P. 567.

"Ego tandem advocatus *aperui matricis orificium.*"—P. 567.

"Nonnunquam enim mulieribus *in partu aquam ingenti copia profluxisse vidimus,* aliquando etiam geminam: obstetrices nostræ By-waters nominant."—P. 578.

These are sufficient to shew that Harvey performed many of those manual operations, which are now mostly consigned to the care of the accoucheur.

The first edition of Harvey's work, *de Generatione Animalium, &c.* was published at London, in 4to, 1651, with a dedication from Sir George Ent, to the College of Physicians. Another edition, in English, was published in London, in 1653, but by whom translated does not appear. It is, however, a pretty exact translation from the Latin; and might, perhaps, be published under the auspices of Dr. Ent. It is now, I believe, rather a scarce book. The title runs thus: "Anatomical Exercitations, concerning the Generation of Living Creatures: To which are added, Particular Discourses, of Births, and of Conceptions, &c. By William Harvey,

Harvey, Doctor of Physick, and Professor of Anatomy and Chirurgery, in the Colledge of Physitians of London. 1653."

I remain, with respect, Gentlemen,

Your occasional correspondent,

*A Contributor of some Articles to the Collectanea Medica.*

*May* 6, 1812.

